# Experimental Hyperglycemia Alters Circulating Concentrations and Renal Clearance of Oxidative and Advanced Glycation End Products in Healthy Obese Humans

**DOI:** 10.3390/nu11030532

**Published:** 2019-03-01

**Authors:** Ryan K. Perkins, Edwin R. Miranda, Kristian Karstoft, Paul J. Beisswenger, Thomas P. J. Solomon, Jacob M. Haus

**Affiliations:** 1 School of Kinesiology, University of Michigan, Ann Arbor, MI 48109, USA; ryperkin@umich.edu (R.K.P.); edwinray@umich.edu (E.R.M.); 2 Centre of Inflammation and Metabolism and Centre for Physical Activity Research, Rigshospitalet, University of Copenhagen, DK-2100 Copenhagen, Denmark; Kristian.Karstoft@regionh.dk; 3 Geisel School of Medicine, Dartmouth College, PreventAGE Healthcare, 16 Cavendish Court, Lebanon, NH 03766, USA; pjb@preventagehealthcare.com; 4 School of Sport, Exercise, and Rehabilitation Sciences, College of Life and Environmental Sciences, University of Birmingham, Birmingham, West Midlands B15 2TT, UK; t.solomon@bham.ac.uk; 5 Institute of Metabolism and Systems Research (IMSR), College of Medical and Dental Sciences, University of Birmingham, Birmingham, West Midlands B15 2TT, UK

**Keywords:** RAGE, soluble RAGE, inflammation, hyperglycemia

## Abstract

The purpose of this investigation was to evaluate the effects of experimental hyperglycemia on oxidative damage (OX), advanced glycation end products (AGEs), and the receptor for AGEs (RAGE) through an in vivo approach. Obese subjects (*n* = 10; 31.2 ± 1.2 kg·m^−2^; 56 ± 3 years) underwent 24 h of hyperglycemic clamp (+5.4 mM above basal), where plasma at basal and after 2 h and 24 h of hyperglycemic challenge were assayed for OX (methionine sulfoxide, MetSO, and aminoadipic acid, AAA) and AGE-free adducts (N^e^-carboxymethyllysine, CML; N^e^-carboxyethyllysine, CEL; glyoxal hydroimidazolone-1, GH-1; methylglyoxal hydroimidazolone-1, MG-H1; and 3-deoxyglucosone hydroimidazolone, 3DG-H) via liquid chromatography–tandem mass spectrometry (LC–MS/MS). Urine was also analyzed at basal and after 24 h for OX and AGE-free adducts and plasma soluble RAGE (sRAGE) isoforms (endogenous secretory RAGE, esRAGE, and cleaved RAGE, cRAGE), and inflammatory markers were determined via enzyme-linked immunosorbent assay (ELISA). Skeletal muscle tissue collected via biopsy was probed at basal, 2 h, and 24 h for RAGE and OST48 protein expression. Plasma MetSO, AAA, CEL, MG-H1, and G-H1 decreased (−18% to −47%; *p* < 0.05), while CML increased (72% at 24 h; *p* < 0.05) and 3DG-H remained unchanged (*p* > 0.05) with the hyperglycemic challenge. Renal clearance of MetSO, AAA, and G-H1 increased (599% to 1077%; *p* < 0.05), CML decreased (−30%; *p* < 0.05), and 3DG-H, CEL, and MG-H1 remained unchanged (*p* > 0.05). Fractional excretion of MetSO, AAA, CEL, G-H1, and MG-H1 increased (5.8% to 532%; *p* < 0.05) and CML and 3DG-H remained unchanged (*p* > 0.05). Muscle RAGE and OST48 expression, plasma sRAGE, IL-1β, IL-1Ra, and TNFα remained unchanged (*p* > 0.05), while IL-6 increased (159% vs. basal; *p* > 0.05). These findings suggest that individuals who are obese but otherwise healthy have the capacity to prevent accumulation of OX and AGEs during metabolic stress by increasing fractional excretion and renal clearance.

## 1. Introduction

Diabetes mellitus is an increasingly prevalent public health concern [1]. Alarmingly, an estimated 30 million individuals in the U.S. alone have diabetes [2]. Diabetes may be diagnosed based on an elevated fasting plasma glucose value or the 2 h plasma glucose level after a 75 g oral glucose tolerance test (OGTT) [3,4]. The reduced ability to handle glucose disposal highlights the chronic state of hyperglycemia that individuals with diabetes experience. 

A primary consequence of hyperglycemia is the formation of oxidative damage (OX) and advanced glycation end products (AGEs) [5]. AGEs are long-lasting reactive intermediates formed from nonenzymatic reactions between glucose or other glycating dicarbonyls (i.e., methylglyoxal, glyoxal, and 3-deoxyglucosone) and a target protein, [6,7,8], while OX markers are formed through oxidative processes [9]. Interestingly, the degree of AGE formation is related to glucose concentrations and accessibility to specific protein amine groups (e.g., lysine and arginine). In addition to endogenous production of AGEs and OXs, other factors, such as a diet utilizing high-heat cooking methods [10,11] and renal clearance [12,13,14] contribute to levels of these metabolites. 

Increased circulating levels of AGEs are problematic due to recognition by the receptor for advanced glycation end products (RAGE) and subsequent downstream inflammatory factor and reactive oxygen species production [15,16,17,18]. RAGE signaling can be interrupted in vivo through proteolytic cleavage, forming a cleaved RAGE isoform (cRAGE) [19]. In addition, alternative splicing events lead to the production and cellular expulsion via exocytosis of another RAGE isoform, endogenous secretory RAGE (esRAGE) [20]. Together, cRAGE and esRAGE comprise the totality of solubilized RAGE isoforms (sRAGE). In addition to sRAGE, AGE-related signaling competition may occur through membrane-bound oligosaccharyltransferase 48 (OST48). OST48 is an integral plasma membrane protein that has been identified to bind and transport AGEs from the extracellular to the intracellular compartments for removal [21,22]. The attenuation of AGEs by sRAGE and OST48 suggest levels of these receptors are critical for mitigating AGE–RAGE-mediated inflammation and oxidative stress.

Diabetes is characterized by a state of chronic hyperglycemia, which likely contributes to elevated AGE levels and oxidative stress [23,24,25] and subsequent inflammation [18]. These events are concerning due to their association with well-established health consequences [26,27,28], including vascular complications in type 2 diabetes [29] and inhibition of insulin action in skeletal muscle [30]. Given the link between hyperglycemia and poor health outcomes via the AGE–RAGE axis, it is surprising that the effects of short-term hyperglycemia on this pathway are still relatively unknown [26]. Therefore, the purpose of this study was to evaluate the effects of 24 h of experimental hyperglycemia on the AGE–RAGE axis in the circulation and skeletal muscle (a predominant metabolic tissue) of obese but otherwise healthy humans. We hypothesized that circulating OX and AGE concentrations and skeletal muscle RAGE expression would increase over the course of the 24 h period as a result of the sustained elevation in blood glucose concentration. Due to the regulatory impact of renal function on circulating OX and AGE levels, we also examined renal OX and AGE clearance and fractional excretion rates. 

## 2. Materials and Methods 

### 2.1. Experimental Design

The overarching goal of this investigation was to determine the effect of 24 h of well-controlled experimental hyperglycemia on the AGE–RAGE axis in the circulation, urine, and skeletal muscle. Enrolled subjects represented a subset of a study population previously reported by our group [31,32]. Briefly, subjects were recruited from Copenhagen, Denmark through an internet-based tool (www.forsoegsperson.dk). Following recruitment, participants completed a detailed medical screening evaluation (medical history, physical exam, and blood profile) to assess the following inclusion criteria: age 18–60 years, body mass index (BMI) 20–35 kg·m^−2^, nonsmoking, weight stable (<2 kg in previous 6 months), inactive (<150 min·week^−1^ of structured exercise), free of disease (cardiovascular, hematological, pulmonary, renal, and hepatic), and not insulin-treated. Based on the inclusion criteria, the final study population was 10 healthy, nondiabetic but overweight/obese females and males. This registered study on www.clinicaltrials.gov (NCT01375270) was approved by the Ethics Committee of the Capital Region of Denmark (H-3-2010-127), and all subjects provided informed written consent to participate. 

Blood and skeletal muscle samples were collected in the basal state and after 2 h and 24 h of hyperglycemia. Participants produced two, 24-h urine collections (the basal state and hyperglycemia challenge). To provide insight into the subjects’ underlying health status, a dual-energy X-ray absorptiometry (DEXA) scan to evaluate body composition, incremental cycle ergometer test to determine VO_2_max (i.e., cardiorespiratory fitness), and oral glucose tolerance test (OGTT) to assess insulin sensitivity were conducted.

### 2.2. Protocol

For the experimental hyperglycemia trial, subjects arrived at the laboratory at 8:00 a.m. following an overnight fast. Upon arrival, contralateral antecubital and dorsal hand vein lines were placed. The sampling hand was warmed to ~60 °C to arterialize blood, and a basal blood draw was taken. A priming dose of glucose was infused through the antecubital vein to raise plasma glucose by 5.4 mM above basal. A variable rate glucose infusion was utilized to maintain hyperglycemia, with arterialized blood samples taken every 5 min and analyzed to titrate glucose infusion rates. Subjects remained in bed for the duration of the trial and were provided three isocaloric liquid meals (Resource Complete, Nestle, Switzerland) after the basal blood draw and 4 h and 8 h after initiation of the hyperglycemic clamp. 

### 2.3. Blood and Urine Collection

Blood samples for plasma glucose were collected at regular intervals at bedside into heparinized syringes and analyzed immediately via the glucose oxidase approach (ABL 725; Radiometer, Copenhagen, Denmark). Blood for sRAGE, inflammation, OX and AGE products, and cystatin C analysis were collected at baseline and 2 h and 24 h of hyperglycemia. Samples were immediately placed on ice and centrifuged at 3500× *g* for 15 min at 4 °C, aliquoted, and stored at −80 °C. HbA1c was determined by high-performance liquid chromatography (Tosoh G7 Analyzer, San Francisco, CA, USA). Two, 24-h urine collections (before and after the hyperglycemia trial) were completed for OX marker, AGE, creatinine, and isoprostane concentration.

### 2.4. sRAGE, Inflammation, and Urine Analyses

Total plasma sRAGE was measured via enzyme-linked immunosorbent assay (ELISA; DRG00, R&D Systems, Minneapolis, MN, USA). This total sRAGE quantification approach included the cleaved (cRAGE) and endogenous secretory (esRAGE) isoforms. To quantify plasma esRAGE specifically, another ELISA was utilized (K1009-1AS, One International, Mountain View, CA, USA). Plasma cRAGE was calculated by subtracting esRAGE from total sRAGE [33,34,35]. Proinflammatory cytokines (IL-6, IL-1β, and TNFα) were analyzed in duplicate by 5-plex electrochemiluminescence immunoassay (MSD, Rockville, MD, USA). IL-1Ra was measured using a Quantikine ELISA Kit (R&D Systems). Urinary creatinine was measured via absorption photometry (Cobas 8000, Roche Diagnostics, Basel, Switzerland), and free 8-iso prostaglandin F2α (8-iso PGF2α), a marker for oxidative stress [36], was assessed in triplicate using an 8-isoprostane ELISA (Cayman Chemicals, Ann Arbor, MI, USA). To evaluate urine glucose concentration [37], 24-h urine collections were assessed via the glucose oxidase colorimetric method (MAK263, Sigma-Aldrich, St. Louis, MO, USA).

### 2.5. OX Marker and AGE Analyses

As previously described [27,38,39], OX markers (methionine sulfoxide, MetSO, and aminoadipic acid, AAA) and AGE-free adducts (N^e^-carboxymethyllysine, CML; N^e^-carboxyethyllysine, CEL; 3-deoxyglucosone hydroimidazolone, 3DG-H; glyoxal hydroimidazolone-1, GH-1; and methylglyoxal hydroimidazolone-1, MG-H1) were measured in plasma and urine. Analysis was performed in a blinded fashion using internal stable heavy isotope standards (PreventAGE Healthcare Technology, Hanover, New Hampshire). Briefly, sample ultrafiltrate (10 K cutoff Amicon filter) were analyzed by liquid chromatography–tandem mass spectrometry (LC–MS/MS) using a 6490 Triple Quadrupole MS system with a 1290 Rapid Resolution LC system to detect analytes. OX markers and AGEs were separated using a Waters X-select HSS T3 column with a mobile phase gradient of methanol and water with 0.20% heptafluorbutyric acid. 

### 2.6. eGFR, Fractional Analyte Excretion, and Renal Analyte Clearance

To provide insight into renal handling and elimination of the analytes of interest, estimated glomerular filtration rate (eGFR) was calculated from plasma cystatin C (DSCTC0, R&D Systems, Minneapolis, MN, USA) using the CKD-EPI (Chronic Kidney Disease Epidemiology Collaboration) equation [40]. eGFR was used to calculate fractional OX marker and AGE excretion (FE), as previously described [13], using the following formula:

FE (%) =([urine analyte] × 24 h urine volume × 100) ÷([plasma analyte] × eGFR × 1440)

To complement FE, renal clearance (RC) of OX markers and AGEs was also calculated as before [12] using the following formula:

$$RC\;\left(\mathrm{mL}\cdot \min^{-1}\right)\;=\left[urine\;analyte\right]\;\times \;24\;h\;urine\;volume\;÷\left[plasma\;analyte\right]\;\times \;1440$$

### 2.7. Skeletal Muscle Biopsy

A skeletal muscle biopsy [41,42,43,44] of the m. vastus lateralis was obtained basally and after 2 h and 24 h of hyperglycemia under local anesthetic (Lidocaine HCL, 1%). Following each muscle biopsy, excess blood, visible fat, and connective tissue were removed, and the muscle sample was immediately flash-frozen in liquid nitrogen and stored at −80 °C until analysis.

### 2.8. Immunoblotting

RAGE and OST48 protein expression in skeletal muscle were quantified via Western blot analysis. Approximately 10 mg (wet weight) of frozen muscle tissue was homogenized by ceramic beads (lysing matrix D, FastPrep-24 homogenizer, MP Biomedical, Santa Ana, CA, USA) in 20 volumes of ice-cold buffer made with 150 nM NaCL, 1 mM Na_2_ EDTA, 1 mM EGTA, 2.5 mM NA pyrophosphate, 1 mM β-glycerophosphate, 1 mM Na_3_VO_4_, 1% Triton, and 1 μg·mL^−1^ leupeptin (Cell Signaling Technology, Beverly, MA, USA) with an added protease and phosphatase inhibitor cocktail (MS-SAFE; Sigma Aldrich, St. Louis, MO, USA). Total protein concentration was determined via BCA assay (Pierce Biotechnology, Rockford, IL, USA). Equal protein was loaded on a gradient gel (BioRad, Hercules, CA, USA) and resolved using SDS-PAGE, transferred to a nitrocellulose membrane, and blocked with Odyssey Blocking Buffer (LI-COR Biosciences, Lincoln, NE, USA) in TBS for 1 h at room temperature. RAGE (1:500, AB3611, Abcam, Cambridge, MA, USA) and OST48 (1:1000, SC74408, Santa Cruz, CA, USA) primary antibody incubations took place overnight, rocking, at 4 °C. GAPDH (1:5000, 14C10, Cell Signaling, Beverly, MA, USA) served as a loading control and was incubated for 1 h at room temperature while rocking. Secondary antibody (LICOR Biosciences, Lincoln, NE, USA) incubations occurred for 1 h at room temperature while rocking. Protein expression (RAGE, OST48, and GAPDH) was visualized with a NIR system (Odyssey, LICOR Biosciences, Lincoln, NE, USA) and quantified using Image Studio (LICOR Biosciences, Lincoln, NE, USA). In accordance with the manufacturers’ guidelines, RAGE was detected as two distinct bands representing the pre- (43 kDa) and post- (48 kDa) glycosylation states. Therefore, expression of both bands was summed to represent total RAGE expression. GAPDH expression was validated against total protein and verified to be stable throughout the experimental period (basal: 0.0050 ± 0.0009; 2 h: 0.0041 ± 0.0003; 24 h: 0.0048 ± 0.0008 AU (arbitrary units); *p* > 0.05).

### 2.9. Statistical Analyses

Data were analyzed via IBM SPSS v24 (SPSS, Chicago, IL, USA). A one-way analysis of variance (ANOVA) with repeated measures was used to compare protein expression (RAGE and OST48) and circulating OX marker/AGE/inflammation/sRAGE concentrations among the three time points (basal, 2 h, and 24 h), and a Bonferroni post hoc test was utilized to examine specific differences when appropriate. The Student’s *t*-test was used to compare urine OX markers and AGEs, eGFR, renal clearance, and fractional analyte excretion between baseline and 24 h of the experimental condition. Significance was set at *p* < 0.05. Data are presented as mean ± SE.

## 3. Results

### 3.1. Subjects Characteristics

The subject characteristics are presented in Table 1 and have been previously published [31,32]. These data are presented here for context to the current study’s main objectives. Female and male participants were matched with regard to age, BMI, and VO_2_max. As portrayed in Table 1, these participants were considered normoglycemic according to the fasting glucose, 2 h glucose OGTT, and HbA1c values [4,45]. Aside from being overweight or obese, participants were in good health as highlighted by a high absolute VO_2_max, an indicator of cardiorespiratory fitness (female: >90th percentile; male: >80th percentile) [46]. 

### 3.2. OX Markers and AGEs

Plasma concentrations of OX markers and AGEs measured basally and 2 h and 24 h after the hyperglycemic challenge are presented in Table 2. In general, most free adducts were reduced during the hyperglycemic period. MG-H1 decreased (*p* < 0.05) at 2 h (−40%) and 24 h (−47%), while MetSO (−34%), AAA (−37%), CEL (−24%), and G-H1 (−18%) decreased by 24 h (*p* < 0.05). CML exhibited a differential response as plasma levels increased 72% (*p* < 0.05) by 24 h. Urine concentration of OX markers and AGEs were unchanged (*p* > 0.05) at 24 h (Table 2). Basal urine analyte concentrations were 46–196% (6 of the 7 free adducts were 178–196%) higher than the plasma.

### 3.3. eGFR, Fractional Excretion, and Renal Clearance of OX Markers and AGEs

**Figure 1:**
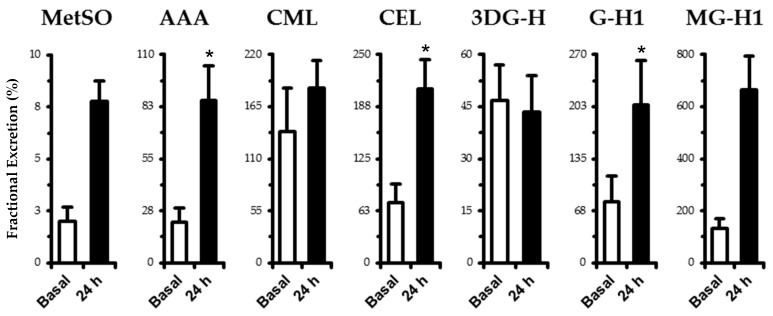
Fractional excretion of oxidation (MetSO and AAA) and AGE-free adducts (CML, CEL, 3DG-H, GH-1, and MG-H1) in human plasma. Samples were taken in the basal state following an overnight fast and after 24 h of experimental hyperglycemia (+5.4 mM above basal plasma glucose levels). See fractional excretion in Materials and Methods for computational details. Data are mean ± SE. * *p* < 0.05 vs. basal.

No differences (*p* > 0.05) were found in eGFR between basal (102 ± 5 mL·min^−1^) and 2 h (110 ± 6 mL·min^−1^) and 24 h (111 ± 4 mL·min^−1^) of experimental hyperglycemia. Fractional excretion of measured analytes is shown in Figure 1. MetSO (+5.8%), AAA (+64%), CEL (+135%), G-H1 (+127%), and MG-H1 (+532%) increased (*p* < 0.05) at the 24 h time point. Fractional excretion of CML and 3DG-H remained unchanged (*p* > 0.05). Renal clearance generally followed the same trend as fractional excretion. MetSO (basal: 1.93 ± 0.63, 24 h: 8.67 ± 1.16 mL·min^−1^), AAA (basal: 20.7 ± 6.0, 24 h: 94.0 ± 19.4 mL·min^−1^), and G-H1 (basal: 81.1 ± 30.6, 24 h: 216.3 ± 51.8 mL·min^−1^) increased (*p* < 0.05), while 3DG-H (basal: 46.6 ± 9.6, 24 h: 46.7 ± 10.5 mL·min^−1^), MG-H1 (basal: 147 ± 45, 24 h: 198 ± 49 mL·min^−1^), and CEL (basal: 70.7 ± 19.9, 24 h: 62.3 ± 14.2 mL·min^−1^) remained unchanged (*p* > 0.05) and CML (basal: 134 ± 38, 24 h: 56 ± 13 mL·min^−1^) decreased (*p* < 0.05).

### 3.4. Plasma and Urine Biomarkers

Plasma inflammatory factors, total sRAGE, and sRAGE isoforms are presented in Table 3. IL-6 increased in a stepwise fashion (24 h vs. 2 h: 80%, *p* < 0.10; 24 h vs. basal: 159%, *p* < 0.05). IL-1β tended (main effect, *p* < 0.10) to increase over time (2 h vs. basal: 112%; 24 h vs. basal: 558%), while TNFα and IL-1Ra remained unchanged (*p* > 0.05). Urinary isoprostane (basal: 549 ± 111; 24 h: 1890 ± 268 pg·mg creatinine^−1^) and glucose concentrations (basal: 0.031 ± 0.017; 24 h: 0.226 ± 0.040 mM) increased 328% and 629% (*p* < 0.05), respectively. Total sRAGE and both sRAGE isoforms (esRAGE and cRAGE) remained unchanged throughout the 24 h period (*p* > 0.05).

### 3.5. Skeletal Muscle RAGE and OST48

**Figure 2:**
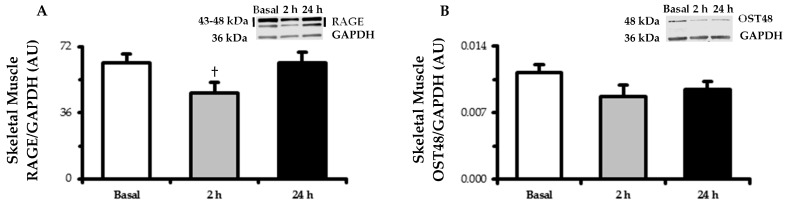
Skeletal muscle (m. vastus lateralis) protein expression determined by Western blot of the (**A**) RAGE and (**B**) OST48. Biopsy-derived muscle samples were taken in the basal state following an overnight fast and after 2 h and 24 h of experimental hyperglycemia (+5.4 mM above basal plasma glucose levels). Representative blot images are embedded within their respective data figures. RAGE bands (43 and 48 kDa) were quantified together. GAPDH served as a loading control. Data are means ± SE. † *p* < 0.10 vs. basal.

RAGE protein expression (Figure 2) tended to decrease (*p* < 0.10) in muscle at 2 h (−23%) and returned to baseline by 24 h. OST48 expression followed a similar tendency as RAGE by nonsignificantly decreasing (−18%, *p* > 0.05) at 2 h. 

## 4. Discussion

The primary goal of this investigation was to evaluate the effects of experimental hyperglycemia on the AGE–RAGE axis in humans. The major findings from this paper are that, after 24 h of experimental hyperglycemia, (1) most circulating OX and AGE-free adduct concentrations decreased, (2) OX and AGE-free adduct clearance and fractional excretion generally increased, and (3) CML behaved differentially than the other measured analytes, whereby the circulating concentration increased and clearance and fractional excretion failed to compensate. Given that production of OX and AGEs due to metabolic stress typically increases [5], these findings were surprising and divergent from our original hypothesis and potentially reflective of the participants’ good overall health status enabling compensation for the metabolic load.

Formation of OX and AGEs results from a complex cascade of reactions producing this heterogeneous group of compounds that are intertwined and capable of eliciting numerous negative health outcomes. Glucose and other reactive dicarbonyls stimulate the formation of OX and AGEs [6,7,8]; therefore, individuals with type 2 diabetes possessing higher basal circulating concentration of these analytes is not surprising [24,25,47,48]. Furthermore, pharmacological reduction of postprandial hyperglycemia lowers circulating AGE load [13,23]. Given this context, data presented here demonstrating a decrease in circulating concentration of most OX (MetSO and AAA) and AGEs (CEL, G-H1, and MG-H1) was unexpected, and the magnitude of these decreases was impressive, ranging from 18–47% by 24 h of the hyperglycemic challenge.

To our knowledge, few diabetes-related studies have investigated the effects of exogenous sources of energy on circulating reactive dicarbonyl and AGE load [23,47,48]. These studies are unique and generally in agreement as an OGTT and mixed meal increased circulating reactive dicarbonyls in patients with type 1 [23] and type 2 diabetes [47,48], respectively. While these studies provide great insight into mechanisms driving AGE formation, they differ from the current study in many ways. Perhaps most importantly, the current study included generally healthy participants, whereas the focus of other studies was individuals type 1 [23] and type 2 diabetes [47,48]. Furthermore, the current study measured AGE-free adducts with a hyperglycemia-induced glucose clamp (i.e., intravenous glucose administration) with mixed liquid meals, whereas the previous studies measured dicarbonyl intermediates following an oral glucose consumption (i.e., OGTT) [48] or a mixed meal only [23,47]. Therefore, it is likely that the differences in underlying health status, route and/or composition of energy consumption, and specific analytes assessed explain the differential findings between the previously published studies and the current study.

Many factors contribute to OX and AGE accumulation. Renal clearance is critical [12,13] and typically sufficient to maintain homeostatic OX and AGE levels, countering endogenous production and exogenous ingestion. In support, we found that many circulating OX and AGEs exhibited a strong inverse relationship with renal clearance rates (data not shown). The effect of renal function on clearance of AGEs is highlighted by robust accumulation of AGEs in the circulation of patients with renal failure [12] and a decrease in AGE burden after kidney transplantation [49,50]. Chronic AGE load modifies structural properties of proteins and intrinsic cellular functioning through extracellular matrix cross-linking [8,51,52]. For example, five months of CML-modified albumin injection into healthy rats (eliciting similar circulating AGE levels observed in individuals with diabetes) resulted in a 50% increase in renal AGE content and histological features indicative of glomerulosclerosis [53]. These types of cellular outcomes have profound implications for vascular and renal processes and likely underscore the impaired AGE clearance observed in patients with renal failure. 

In general, renal handling of OX and AGEs did not appear to be impaired in this study as basal clearance and excretion were similar to other human studies [12,13] and clearance of OX and AGEs (i.e., MetSO, AAA, CEL, G-H1, and MG-H1) increased substantially in response to the hyperglycemic challenge. This large increase in OX and AGE removal through the kidneys likely explains the unexpected finding of reduced circulating levels of most OX and AGEs following the challenge. Information regarding factors that impact renal handling of OX and AGEs is scarce, and these factors seem to differ among metabolites [54]. It is believed that near-normal renal health is vital to maintaining homeostatic AGE burden [55], which highlights the delicate balance between clearance and OX and AGE accumulation. Participants in this study were in good overall health (i.e., good absolute VO_2_max based on age-related reference norms, normal eGFR, fasting and 2 h glucose); therefore, the kidney function was able to compensate for the potential to increase circulating OX and AGE levels in response to the metabolic stress imparted by 24 h of experimental hyperglycemia. 

Renal handling of CML behaved differentially than the other OX and AGEs, resulting in a dramatic >70% increase in circulating CML concentrations. CML is a predominant circulating AGE, likely due to the multiple routes by which CML is presented to the circulation [56]. In addition, CML has a reported high removal rate through the kidney in healthy humans [12,13] and constitutes a major AGE in renal tissue of diabetic nephropathy [57]. In this study, unlike most other measured OX and AGEs, fractional excretion of CML remained unchanged, while renal CML clearance decreased by over 50%. The inability of the kidney to maintain an appropriately high clearance rate of CML likely accounts for the accumulation of CML in the circulation and may indicate that the kidney preferentially clears other metabolites at the expense of CML. This finding is novel and problematic for AGE burden as it indicates that even healthy kidneys may fail to clear CML at a rate that prevents its accumulation under some circumstances.

In addition to endogenous production of AGEs, the diet also contributes to AGE burden [10,11]. AGEs are naturally present in animal-derived foods, and their production increases in all food types with cooking. More specifically, cooking methods utilizing high heat (e.g., grilling, broiling) stimulate the Maillard browning reaction, which leads to the formation of new AGEs [58,59]. For logistical reasons, each participant received three liquid meals (Resource Complete). These meals were milk-based, which is reportedly low in AGE content (comparable to raw fruits and vegetables) [59]. Therefore, it is not likely that diet-induced contribution to circulating AGEs during this study was appreciable; however, we cannot discount this possibility. Data exists suggesting a link between dietary CML intake and route of elimination (i.e., urinary vs. fecal). It is possible that these small meals shifted CML removal from the kidney to fecal-based mechanisms of elimination due to the kidney reaching a saturation point, at which point fecal excretion appears to become increasingly important [56,60]. However, fecal-based removal does not appear to be a significant factor in the current study as CML renal clearance did not increase to a “saturation point” but rather decreased by over 50%. Few studies have focused on elimination of AGEs from the body, and those that do, tend to emphasize clearance of only a single AGE. Further research on the interaction between different AGEs and their associated clearance is needed to clarify these important questions.

The overall outcome of activating the AGE–RAGE axis is inflammation and reactive oxygen species production [15,16,17,18]. Herein, we showed that skeletal muscle RAGE expression tended to decrease by 2 h and returned to basal levels after 24 h of the hyperglycemic challenge. This is an interesting finding as hyperglycemia induces RAGE expression in cell culture models [61]. One explanation for this observation is that decreased total circulating AGEs attenuated RAGE signaling at the tissue level, thereby temporally lowering expression of RAGE as production of the RAGE receptor works in a positive feedback manner through NF-κB [17]. RAGE signaling can also be interrupted in vivo through competitive binding. Membrane-bound OST48 (i.e., AGER1) [22] and sRAGE isoforms also bind AGEs but act to suppress the inflammatory response. The latter would suggest it is advantageous to maintain high sRAGE levels [33,62,63]. For example, we have shown that sRAGE isoforms are attenuated across the glucose-tolerance continuum [33], that weight loss is related to increased sRAGE [64], and that a single high-fat meal reduces sRAGE levels [34]. In this study, muscle OST48 expression, total sRAGE, and both sRAGE isoforms (cRAGE and esRAGE) remained unchanged. Additionally, the circulating inflammatory response was modest. IL-1β increased a nonsignificant ~100% and IL-6 reached significance by increasing over 100% by 24 h. It is possible that this inflammatory response was initiated by increasing CML and remained modest due to the other OX and AGEs decreasing or remaining unchanged.

This study is not without limitations. Because diet can influence AGE levels [10,11], it is possible that the small meals influenced circulating concentrations. The effect of the provided meals on OX and AGE levels are likely minimal due to the contents being dairy-based [59]. Intravenous fluids (along with the liquid meals) were also administered to the participants over the 24 h experimental periods. Though necessary for study logistical purposes, it is likely that providing fluids impacted OX and AGE circulating concentrations by influencing excretion. In fact, it has been previously observed that aggressively administering fluids over 5 h reduced circulating OX and AGEs in Pima Indians (personal communication via Dr. Paul Beisswenger [65]), though to a smaller degree than in the current study (Pima Indians: ~15–30% vs. current study: ~18–47% decrease). In addition, though urine glucose output remained low (basal: 0.031; hyperglycemia: 0.226 mM), it did increase during the hyperglycemic challenge. Because plasma glucose levels were experimentally increased to near renal glucose resorption threshold (~10.5 mM), it is possible that the small amount of glucose excreted in the urine contributed to some osmotic diuresis. 

## 5. Conclusions

In conclusion, this study provides a targeted, yet comprehensive analysis of the effects of experimental hyperglycemia on the AGE–RAGE axis using an in vivo approach in humans. The major findings from this study demonstrate that most circulating OX and AGE concentrations decrease in response to an acute hyperglycemic state, likely due to the kidneys’ capacity to increase clearance and fractional excretion. However, a consequence of increased removal of these metabolites may be the accumulation of circulating CML due to reduced renal clearance capacity. Because AGEs are ubiquitous and exhibit subtle differences, future studies should focus on both interactive cellular effects and clearance capacities of AGEs.

## Figures and Tables

**Table 1:** Subject characteristics.

Variable	Participants
*N* (Female/Male)	10 (2/8)
Age (years)	56 ± 8
Height (m)	1.74 ± 0.03
Body mass (kg)	95.8 ± 6.3
BMI (kg·m^−2^)	31.3 ± 1.2
Body fat (%)	36.1 ± 1.8
VO_2_max (L·min^−1^)	3.0 ± 0.1
VO_2_max (mL·kg^−1^·min^−1^)	32.3 ± 1.6
HbA1c (mmol·mol^−1^)	37.7 ± 1.1
Fasting glucose (mM)	5.3 ± 0.2
Glucose, 2 h OGTT (mM)	6.7 ± 0.3

Values are mean ± SE. BMI, body mass index; OGTT, oral glucose tolerance test.

**Table 2:** Oxidation markers and advanced glycation end product (AGE)-free adduct concentrations in plasma and urine.

Condition	MetSO (nM)	AAA (nM)	CML (nM)	3DG-H (nM)	CEL (nM)	G-H1 (nM)	MG-H1 (nM)
**Plasma**							
Basal	1120 ± 125	1056 ± 116	74 ± 6	219 ± 17	55 ± 5	10.4 ± 0.9	127 ± 27
2 h	1015 ± 103	870 ± 72	100 ± 13	204 ± 18	49 ± 5	10.0 ± 0.6	58 ± 8*
24 h	689 ± 38*^,#^	625 ± 53*^,#^	123 ± 9*	211 ± 11	40 ± 2*	8.3 ± 0.4*^,#^	50 ± 4*
**Urine**							
Basal	1797 ± 349	18,192 ± 5452	8208 ± 1957	9388 ± 2241	3188 ± 847	713 ± 240	12,278 ± 2199
24 h	1487 ± 172	14,004 ± 2266	6004 ± 601	8509 ± 1080	2165 ± 219	424 ± 86	8684 ± 1382

Values are mean ± SE. MetSO, methionine sulfoxide; AAA, aminoadipic acid; CML, N^e^-carboxymethyllysine; CEL, N^e^-carboxyethyllysine; 3DG-H, 3-deoxyglucosone hydroimidazolone; G-H1, glyoxal hydroimidazolone-1; MG-H1, methylglyoxal hydroimidazolone-1; * *p* < 0.05 vs. basal, ^#^ *p* < 0.05 vs. 2 h.

**Table 3:** Plasma inflammatory marker, total sRAGE, and sRAGE isoform concentrations.

Time	IL-1β (pg·mL^−1^)	IL-1Ra (pg·mL^−1^)	IL-6 (pg·mL^−1^)	TNFα (pg·mL^−1^)	Total sRAGE (pg·mL^−1^)	esRAGE (pg·mL^−1^)	cRAGE (pg·mL^−1^)
Basal	0.101 ± 0.023	254 ± 28	1.064 ± 0.137	15.6 ± 0.8	690 ± 78	214 ± 23	476 ± 56
2 h	0.153 ± 0.043	277 ± 32	1.522 ± 0.247	14.6 ± 0.7	635 ± 70	200 ± 21	436 ± 51
24 h	0.204 ± 0.068	249 ± 25	2.369 ± 0.258 *^,#^	15.3 ± 0.6	658 ± 69	208 ± 21	450 ± 49

Values are mean ± SE. IL-1β, interleukin-1beta; IL-Ra, interleukin 1-receptor antagonist; IL-6, interleukin-6; TNFα, tumor necrosis factor-α; sRAGE, soluble receptor for advanced glycation end products; esRAGE, endogenous secretory receptor for advanced glycation end products; cRAGE, cleaved receptor for advanced glycation end products. * *p* < 0.05 vs. basal. ^#^ *p* < 0.10 vs. 2 h.
